# Dengue Virus Induces the Release of sCD40L and Changes in Levels of Membranal CD42b and CD40L Molecules in Human Platelets

**DOI:** 10.3390/v10070357

**Published:** 2018-07-05

**Authors:** Daniel Núñez-Avellaneda, Manuel Alejandro Mosso-Pani, Luvia E. Sánchez-Torres, María Eugenia Castro-Mussot, Norma Angélica Corona-de la Peña, Ma. Isabel Salazar

**Affiliations:** 1 Departamento de Inmunología, Escuela Nacional de Ciencias Biológicas, Instituto Politécnico Nacional, Unidad Profesional “Lázaro Cárdenas”, Colonia Santo Tomás, Ciudad de México CP 11340, Mexico; denys_8611@hotmail.com (D.N.-A.); qbp.mosso@hotmail.com (M.A.M.-P.); luviasanchez@hotmail.com (L.E.S.-T.); maru278@hotmail.com (M.E.C.-M.); 2 Hospital Carlos McGregor, Instituto Mexicano del Seguro Social, Colonia del Valle, Ciudad de México CP 03100, Mexico; norma_acp@hotmail.com; 3 Laboratorio de Virología e Inmunovirología, Departamento de Microbiología, Escuela Nacional de Ciencias Biológicas del Instituto Politécnico Nacional, Unidad Profesional “Lázaro Cárdenas”, Colonia Santo Tomás, Ciudad de México CP 11340, México

**Keywords:** Immune response, platelets, dengue virus, CD40L

## Abstract

Platelets are considered as significant players in innate and adaptive immune responses. The adhesion molecules they express, including P-selectin, CD40L, and CD42b, facilitate interactions with many cellular effectors. Upon interacting with a pathogen, platelets rapidly express and enhance their adhesion molecules, and secrete cytokines and chemokines. A similar phenomenon occurs after exposure of platelets to thrombin, an agonist extensively used for in vitro activation of these cells. It was recently reported that the dengue virus not only interacts with platelets but possibly infects them, which triggers an increased expression of adhesion molecule P-selectin as well as secretion of IL-1β. In the present study, surface molecules of platelets like CD40L, CD42b, CD62P, and MHC class I were evaluated at 4 h of interaction with dengue virus serotype 2 (DENV-2), finding that DENV-2 induced a sharp rise in the membrane expression of all these molecules. At 2 and 4 h of DENV-2 stimulation of platelets, a significantly greater secretion of soluble CD40L (sCD40L) was found (versus basal levels) as well as cytokines such as GM-CSF, IL-6, IL-8, IL-10, and TNF-α. Compared to basal, DENV-2 elicited more than two-fold increase in these cytokines. Compared to the thrombin-induced response, the level generated by DENV-2 was much higher for GM-CSF, IL-6, and TNF-α. All these events induced by DENV end up in conspicuous morphological changes observed in platelets by confocal microscopy and transmission electron microscopy, very different from those elicited by thrombin in a more physiological scenery.

## 1. Introduction

Platelets not only participate actively in hemostasis, but also in the function of the endothelium and maintaining vascular integrity [1]. In addition, they play a significant role in the immune response. For example, they have both pre-mRNA and mature IL-1β, making them important producers of this interleukin [2]. They also contain some cytokines and chemokines (e.g., RANTES, MIP-1α and TGF-β) [3] and present antigens through MHC class I molecules [4].

Among other immunological activities of platelets is their notable capacity to bind to several viruses, including human immunodeficiency virus, rotavirus, hepatitis C virus, cytomegalovirus, Epstein-Barr virus, and dengue virus (DENV) [5,6]. Moreover, platelets exhibit numerous receptors that respond to viruses [7] and release antimicrobial peptides [5,8]. Their interaction with pathogens occurs through membranal receptors, such as Toll-like receptors (TLRs 1, 2, 3, 4, 5, 6, 7/8 and 9) [9], Fc receptors, and DC-SIGN molecules [5].

Furthermore, platelets are the main source of soluble CD40L (CD154) [10], a molecule that plays a pivotal role in modulating innate and adaptive immune responses, mostly through the CD40L-CD40 interaction [11]. Among the different cellular functions made possible through CD40L-CD40 binding are in B lymphocytes, the maturation and isotype switching induced by T cells [12], and, in endothelial cells, the expression of adhesion molecules (e.g., ICAM-1, E-selectin, P-selectin, and VCAM-1) [13,14], and the release of MCP-1, IL-6, and tissue factor [12,15].

In severe dengue cases, the three most important characteristics found are an acute thrombocytopenia, endothelial dysfunction and a cytokine storm. Numerous studies have explored the underlying mechanisms involved in DENV-induced thrombocytopenia, leading to multiple hypotheses [16,17,18]. Furthermore, hemorrhagic clinical outcomes in dengue cases are also accompanied by a significant endothelial activation and dysfunction [19]. Thrombocytopenia is observed in both dengue and dengue hemorrhagic cases, although it is more frequent and profound in the latter ones [20]. Among the mechanisms underlying thrombocytopenia in dengue are: Suppression of marrow that affects both megakaryopoiesis and thrombopoiesis; antibodies against viral protein NS1 self-reacting with platelets components [20]; and apoptosis through platelets’ mitochondrial dysfunction [16]. 

Compared to platelets from healthy donors, those from patients infected with DENV show a decay in their aggregation capacity [21]. Upon contact with DENV, there is P-selectin glycoprotein (CD62P)-mediated activation of platelets as well as a significant release of IL-1β [16]. A characteristic reported in dengue secondary infections is the phosphatidylserine translocation to the platelet membrane, an event that could possibly increase macrophage-mediated phagocytosis and contribute to thrombocytopenia [22]. During a severe dengue outcome, however, the entire mechanisms underlying the dramatic drop in the concentration of platelets and the increased production of the immune mediators are still unclear. The impact of these factors on the overall immune response also requires further investigation.

The aim of this study was to examine the response of platelets to DENV serotype 2 (DENV-2) in an in vitro system. We focus on the protein levels of some immune response effectors (CD40L/MHC class I molecules), such as the adhesion molecules required for intercellular interactions (CD42b and CD62P). Also, soluble effectors such as cytokines (IL-8, IL-6, TNF-α, IL-1-β, IL-10 and GM-CSF) and sCD40L were examined. Finally, the impact of these changes induced by DENV-2 was evaluated at the morphological level in platelets. Possible functional implications for the observed changes are discussed. 

## 2. Material and Methods

### 2.1. DENV-2 Stock Preparation and Titration

The dengue virus stock employed herein was the serotype 2 strain Yuc-20452, isolated in 2012 from a patient in the Yucatan Peninsula, Mexico. DENV-2 was cultivated on C6/36 confluent cell monolayers, propagated in T-75 culture containing L-15 Leibovitz’s medium (Sigma Aldrich, St. Louis, MO, USA), supplemented with 10% of fetal bovine serum (FBS) (Gibco, Waltham, MA, USA), 2 mM L-glutamine, vitamins, and non-essential amino acids. Infections were carried out in a 2% of FBS L-15 Leibovitz’s medium along with an uninfected control treated under the same conditions. After 7 days of infection, the supernatant of virus-infected (DENV group) and uninfected (mock) C6/36 cells was harvested and then both precipitated by using polyethylene glycol 8000 (Sigma Aldrich, St. Louis, MO, USA) to a final concentration of 8%, followed by incubation of the mix overnight at 4 °C. The next day, the solution was centrifuged at 1500 G for 30 min. The pellets were collected and resuspended in phosphate buffer solution (PBS), pH 7.4, and stored at −70 °C to await further use. Virus titration was performed with confluent C6/36 cell monolayers in 24-well plates. Briefly, serial 10-fold dilutions of virus stock were prepared, with 200 µL placed in each well and the plate gently rocked for 40 min at room temperature [3]. Subsequently, cell monolayers were covered with an overlay of semisolid medium consisting of 1% carboxymethyl cellulose (Sigma, St. Louis, MO, USA) in L-15 medium with 2% FBS. After five days, the overlay was gently removed to evaluate the viral antigen by counting the plate units and determining the viral titers with an immunohistochemistry assay. The primary antibody used was a 1:200 dilution of anti-DENV E-protein 4G2, and the secondary antibody used was a 1:2000 dilution of goat anti-mouse IgG-HRP (Invitrogen, Carlsbad, CA, USA). The quantity of infectious particles was expressed as foci-forming units (FFU)/mL, which were revealed with 3,3′ diaminobenzidine (Sigma, St. Louis, MO, USA).

### 2.2. Obtention of Platelets from the Plasma of Healthy Donors 

Platelets were obtained from the plasma of healthy donors who signed informed consent and attended the blood bank of the “Dr. Carlos Mac Gregor Sánchez Navarro” Hospital, belonging to the Instituto Mexicano del Seguro Social, in Mexico City. Blood was drawn into tubes containing a 3.2% citrate solution (Becton Dickinson, Franklin Lakes, NJ, USA), followed by centrifugation of each sample at 400 G for 10 min at RT, which allows the elimination of all the other blood cells and keeps platelet-rich plasmas (PRP). Platelets were separated from the plasma by centrifuging at 1000 G for 10 min at RT. Subsequently, the supernatant was discarded, and the pellet was cautiously re-suspended in Tyrode’s solution (used to resemble physiological conditions), to avoid undesired activation. The platelet suspension was adjusted to 3 × 10^8^/mL and maintained at RT.

### 2.3. DENV-2 Stimulation Assays of Platelets

For the experiments herein described, washed platelet suspensions were adjusted to 1 × 10^7^ cells/mL and incubated with DENV-2 (MOI of 0.2 or 0.5) at 37 °C in 5% CO_2_ atmosphere for 2 or 4 h, depending on each assay. For all the experiments, a mock group (already described) and a positive control with thrombin were included. Briefly, for the cytometric assays, platelets were exposed to a MOI of 0.5 of DENV-2 for 4 h and then analyzed. After treatment, a 100 µL suspension of platelets was transferred to a clean light-protected tube and stained as later described. For ELISA tests, supernatants from DENV-2-treated platelets (MOI = 0.2) were obtained at 4 h and kept at −70 °C until further analysis.

Finally, for examination of platelets by confocal microscopy and transmission electron microscopy (TEM), the initial platelet suspensions were adjusted to 3 × 10^8^ cells/mL and exposed to the virus stimulus (MOI of 0.2 and 0.5, respectively) for not less than 4 h. 

### 2.4. Cytometric Analysis of Molecules in Platelets

To investigate whether DENV-2 induces changes in molecules presented in the membrane of platelets, cytometric assays were carried out for CD62P, CD42b, MHC-I, and CD40L surface molecules. After treatment, the platelets were stained with specific antibodies for 30 min at RT in the dark and cells fixed with 1% paraformaldehyde solution. Antibodies used for these assays were conjugated to fluorophores as follows: anti CD62P-PE (eBiosciences, San Diego, CA, USA), anti CD42b-PE (BioLegend, San Diego, CA, USA), anti MHC-I-FITC (BD Biosciences, San Jose, CA, USA), and anti CD40L-PE (BD Biosciences, San Jose, CA, USA). For each treatment, 1 μg of fluorescent antibody was added to each 10^6^ platelets for incubation. Then platelets were washed and re-suspended in 250 μL of PBS for acquisition. The events corresponding in size (FS) and granularity (SS) to platelets were selected. Readings were made with a FACS ARIA-III (BD, San Jose, CA, USA) cytometer. A total of 150,000 events were acquired and platelets were selected as singlets. The assays were independently conducted in triplicate. Finally, the acquisition analysis was done on FlowJo 8.7 software (BD, Franklin Lakes, NJ, USA).

### 2.5. Evaluation of sCD40L Levels in Platelet Supernatants by ELISA Test

Platelets obtained from the plasma of healthy donors (*n* = 15) were stimulated with DENV-2 (MOI of 0.2) for 2 and 4 h at 37 °C in 5% CO_2_ atmosphere. Thrombin stimulation was used for the positive control. Platelets were centrifuged at 2000 G for 10 min at RT, and the supernatant was collected. Secreted CD40L (sCD40L) was measured with the ab9999-CD40L Human ELISA Kit (Abcam, Cambridge, UK), according to the manufacturer’s instructions. Final readings were made on a Spectra Max M3 (Molecular Devices, San Jose, CA, USA). Assays for each donor were performed in duplicate.

### 2.6. Multi-Array for Cytokine and Chemokine Quantification

For these assays, suspensions of 3 × 10^8^ platelets/mL from heathy donors were stimulated with DENV-2 (0.2 MOI) for 4 h at 37 °C in 5% CO_2_ atmosphere; mock and thrombin (0.02 U/mL) groups were included in the assay. After stimulus, platelets were centrifuged at 2000 G for 10 min at RT; supernatants were collected and kept at −70 °C until used. Supernatants from each experimental group were pooled (*n* = 6 for each pool) for the analysis, and two pools were examined for each experimental group, DENV-2 treated, mock, and thrombin. Cytokines and chemokines (GM-CSF, IFNγ, IL-1α, IL-1β, IL-2, IL-4, IL-6, IL-8, IL-10, IL-12, IL-17A, and TNF-α) were measured in these samples with the Human Inflammatory Cytokines Multi-Analyte ELISArray Kit MEH-004A (Qiagen Hilden, Germany). Readings for optical density were made at OD = 450 nm immediately on a Spectra Max M3 (Molecular Devices, Sunnyvale, CA, USA). The protocol was developed in accordance with the manufacturer’s recommendations. Samples were tested in duplicate, and optical densities for each tested group were graphed.

### 2.7. Analysis of Platelets by Confocal Transmission Microscopy

Platelets were stimulated with a 0.2 MOI for 4 h at 37 °C in 5% CO_2_ atmosphere as well as controls, cells were fixed with 4% paraformaldehyde and analyzed by confocal microscopy to examine broad effects. Images were acquired in bright field on a Nikon Ti Eclipse inverted confocal microscope (Nikon, Tokyo, Japan) through a 100× oil immersion (NA 1.45) objective lens; the transmitted light detector was used as light source. Images were acquired using NIS Elements v.5.00. For transmission electron microscopy analysis, platelets were treated with DENV-2 (0.5 MOI) for 4 h at 37 °C in 5% CO_2_ atmosphere; for these experiments, a mock group and a positive control with thrombin were included (as described in Section 2.1). After treatment, all platelets were fixed with 4% glutaraldehyde and examined under a Joel 2100 transmission electron microscope (JEM-2100F, Tokyo, Japan). For each condition, images were acquired at 50,000×. Assays were independently performed in triplicate. 

### 2.8. Statistical Analysis

The statistical analysis was processed on GraphPad Prism, version 6.0 (GraphPad, San Diego, CA, USA). For comparisons between three groups, one-way ANOVA was used to determine differences. For comparisons between two groups, continuous variables were analyzed with the Student’s *t*-test in case of parametric distributions. The Mann–Whitney U test was employed for nonparametric distributions. 

## 3. Results

### 3.1. Cytometric Analysis for Identification of Platelets

**Figure 1:**
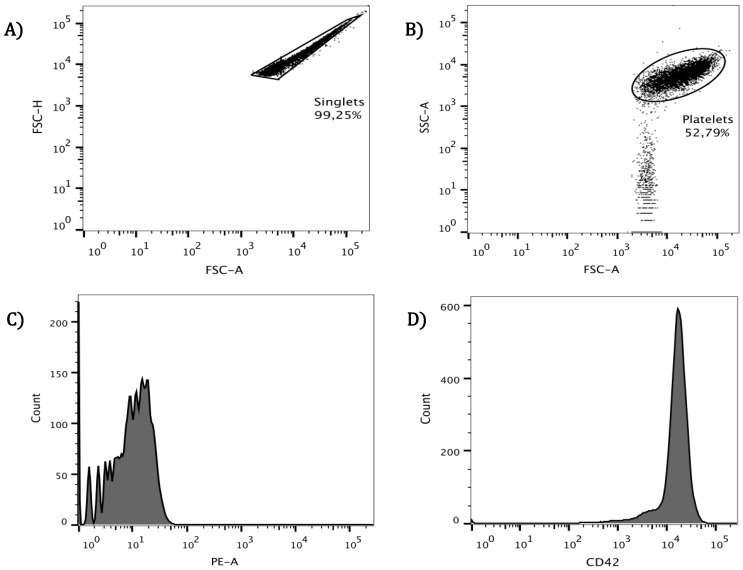
**Platelet selection by cytometric analysis.** Cells were obtained from wash platelet rich plasma (PRP) from healthy donors, carefully washed and resuspended in Tyrode’s solution. (**A**) Washed platelet suspension was acquired in the cytometer and singlets selected; these represented 98 ± 1.25%; (**B**) Platelet population was selected by size and granularity and separated from smaller size particles, which accounted for 52.79% in this particular analysis presented. Finally, to validate the selected population, the CD42b molecule was used as platelet marker, auto-fluorescence was measured (**C**); and anti-CD42-PE identified platelets, which exhibit this marker (**D**).

After carefully eliminating plasma by washing healthy donor platelets, these were analyzed with a flow cytometer. Singlets selected for this purpose (Figure 1A) constituted 98 ± 1.25% of all cells presented in washed suspensions. The singlets were delimited and separated from smaller particles based on size and complexity (as shown in Figure 1B), and that population representing 52.79% of the initial singlets was further examined. To assure that the resulting population corresponded to platelets, the CD42b molecule was used as a specific marker and its presence confirmed on the cell surface. Later, this molecule was also used as an activation indicator in the platelet surface. In the histogram, a unique CD42b-stained population was observed (Figure 1D) and was clearly separated from the auto-fluorescence control (Figure 1C). This type of verification for the platelet population was performed along the subsequent assays.

### 3.2. DENV-2 Induced Density of CD42b and CD62P Adhesion Molecules in Human Platelets

**Figure 2:**
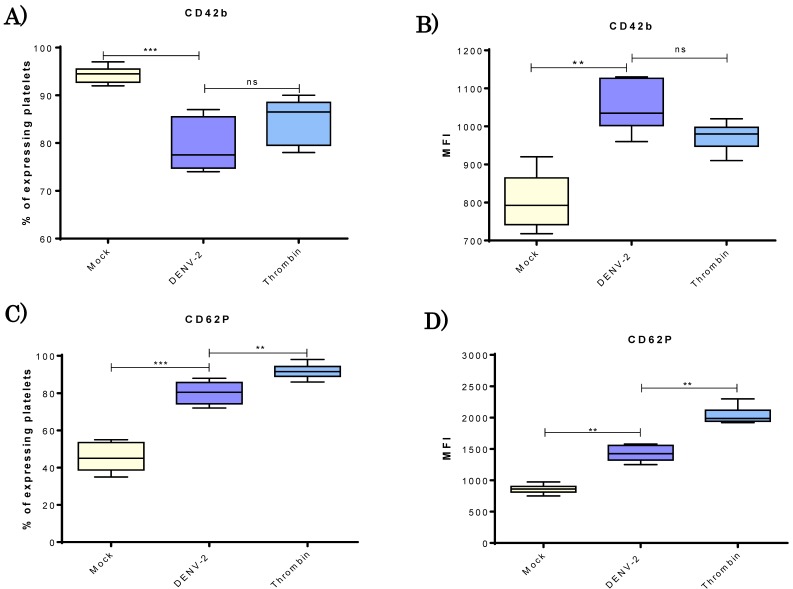
**DENV-2 induced changes in CD42b molecule and activation CD62P marker on human platelets.** Platelets from healthy donors (*n* = 10) were analyzed. Platelets were incubated with DENV2 to a MOI of 0.5 for 4 h at 37 °C. For each experiment, mock and thrombin groups were included. Percentages of platelets expressing CD42b molecule in each group are shown (**A**); as well as median fluorescence intensity (MFI) that indicate density of of CD42b molecule on the cell surface (**B**); as a measure of platelet activation, the percentage of CD62P-expressing platelets was determined (**C**); as well as MFI of CD62P expression (**D**). Unpaired *t*-test and Mann-Whitney U distribution were used for these experiments, where ** *p* < 0.01 and *** *p* < 0.001, ns = not significant.

After platelets treatment with virus, a lower percentage of platelets expressing CD42b (receptor for vWF) was found in the DENV-2-treated than mock groups (73–87% versus 92–98%, respectively; Figure 2A). However, for those DENV-2-treated platelets positive to CD42b, there was a significantly greater density of this molecule in membrane than in the mock group (Figure 2B).

The activation of platelets caused by exposure to DENV-2 at a MOI of 0.5 was assessed by quantifying the percentage of these cells expressing CD62P (P-selectin) and its density on the cellular surface. A significantly higher percentage (74–86%) of DENV-2-exposed platelets tested positive to CD62P than that found in the mock control group (33–55%). The DENV-2 value was close to the 90 ± 5% found in the positive control group (Figure 2C).

### 3.3. DENV-2-Exposed Platelets Released Higher Levels of GM-CSF, IL-6, IL-8, IL-10 and TNF-α.

**Figure 3:**
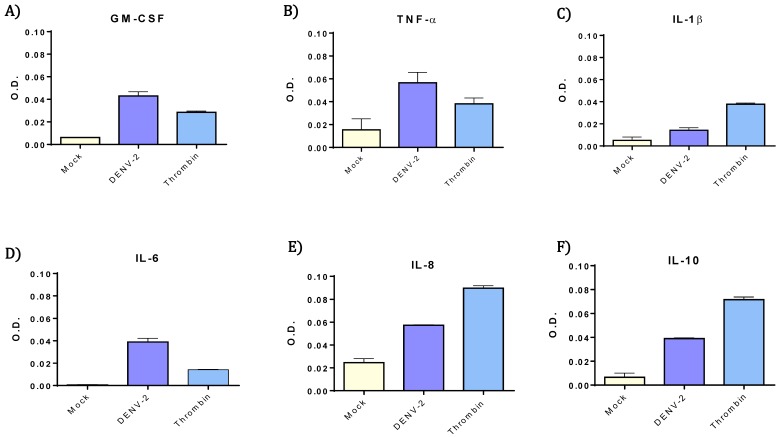
**Higher levels of TNF-α, IL-6, IL-8, GM-CSF, and IL-10 were released from DENV-2 exposed platelets.** Cytokines and chemokines were evaluated, two pools from six supernatants for each experimental group. Original supernatants were obtained from 3 × 10^8^/mL human platelets after 4 h of stimulation with DENV-2 (MOI 0.2), mock or thrombin. Cytokines were measured by optical density GM-CSF (**A**); TNF-α (**B**); IL-1β (**C**); IL-6 (**D**); IL-8 (**E**) and IL-20 (**F**) at 450 nm. TNF-α, IL-8, GM-CSF, and IL-10 were elevated after DENV-2 incubation. IL-6 showed to be more elevated than thrombin control, and IL-1β only showed a small increment. Since each bar contains values of only two pools, only standard deviation is shown. No statistical analysis was performed on these data.

The evaluation of cytokines released by platelets was carried out after 4 h of treatment with DENV-2, using a MOI of 0.2. All the cytokines in multi-analyte-array were analyzed, but only those cytokines that exhibited higher levels that mock control are presented (Figure 3). Among the cytokines that showed increased levels in the resulting supernatants were GM-CSF, IL-6, IL-8, IL-10, and TNF-α, when compared with the mock group. Meanwhile, comparing positive control, the DENV-2-stimulated platelets showed considerably higher levels of GM-CSF, IL-6, and TNF-α than the thrombin-treated platelets.

### 3.4. DENV-2 Induced a Higher Display of MHC Class I Molecules in Platelets

**Figure 4:**
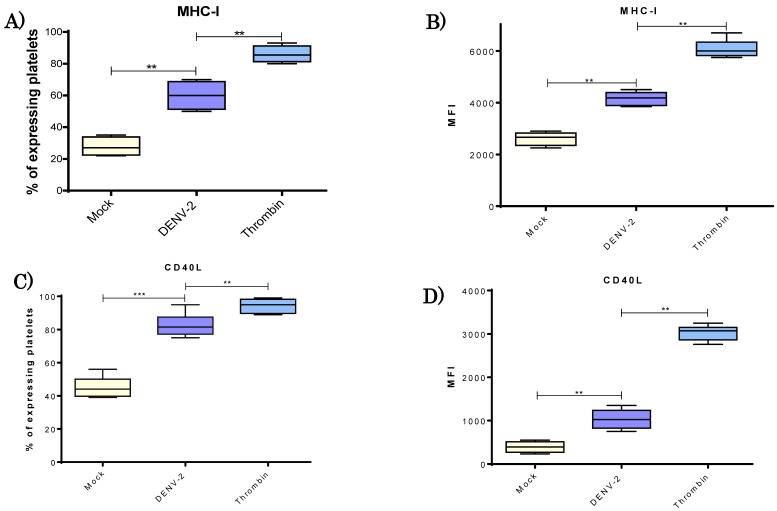
**DENV-2 induced an increase of MHC-I and CD40L molecules on platelets.** Platelets were obtained from healthy donors (*n* = 10) and divided in the three experimental groups. Platelets, DENV-2, and MOI 0.5 were incubated for 4 h at 37 °C; mock and thrombin groups were also processed. Percentage of MHC-I expressing platelets (**A**) and MFI of MHC-I (**B**) were determined. Additionally; percentage of platelets expression CD40L (**C**); and MFI in platelets of CD40L (**D**) were evaluated. Significant increases of MHC class I and CD40L were observed in the DENV-2 treated platelets for both percentages and density of molecules in the cell surface. Unpaired t test and Mann-Whitney U distribution were used, where ** *p* < 0.01 and *** *p* < 0.001).

To explore the relevance of MHC class I molecules in response to DENV-2 in our system, measurement of the intensity of membrane expression was made, as well as the percentage of platelets displaying the respective molecule (Figure 4). There was a significant increase in the percentage of platelets expressing MHC class I in the DENV-2 versus mock group. A range of 23–35% was determined for the mock group and 50–75% for the DENV-2-exposed platelets (Figure 4A). Mean fluorescence intensity (MFI) revealed also a significantly greater mean density (*p* < 0.01) of MHC-I molecules on the surface of DENV-2 stimulated platelets (Figure 4B).

### 3.5. DENV-2 Induced Membranal Display of CD40L and Release of Its Soluble Form in Platelets

**Figure 5:**
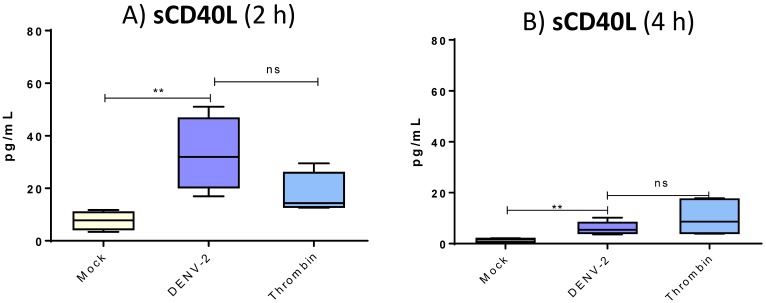
**Human platelets release sCD40L after DENV stimulus.** In supernatants of 3 × 10^8^/mL treated platelets from healthy donors (*n* = 15), soluble CD40L was measured by ELISA test. At 2 and 4 h, platelets were DENV-2 stimulated, MOI 0.2, at 37 °C. Optical density was read at 450 nm and amount of sCD40L calculated for each experimental group. Unpaired t test was used, ** *p* < 0.01 and ns = not significant.

To examine CD40L in platelets in response to DENV-2, we performed both cytometric analysis of membranal form and ELISA test for the soluble molecule. There was a significantly higher percentage of platelets expressing CD40L (mock, 40–55%; DENV-2, 72–90%; thrombin, 92–98%) (Figure 4C) and a greater mean density on the surface of CD40L-positive platelets treated with DENV-2 in comparison with the mock group (MFI; *p* < 0.01) (Figure 4D). Platelets exposed to thrombin served as the positive control and exhibited higher membranal levels of CD40L. Platelets exposed to DENV-2 rapidly released soluble CD40L (Figure 5); at 2 h, levels ranged from 20 to 50 pg/mL. These levels were higher than those released after treatment with thrombin. At 4 h, post-treatment levels of sCD40L in platelets decayed, although they remained significantly higher than the mock group (*p* < 0.001).

### 3.6. DENV-2-Induced Significant Morphological Changes in Human Platelets

**Figure 6:**
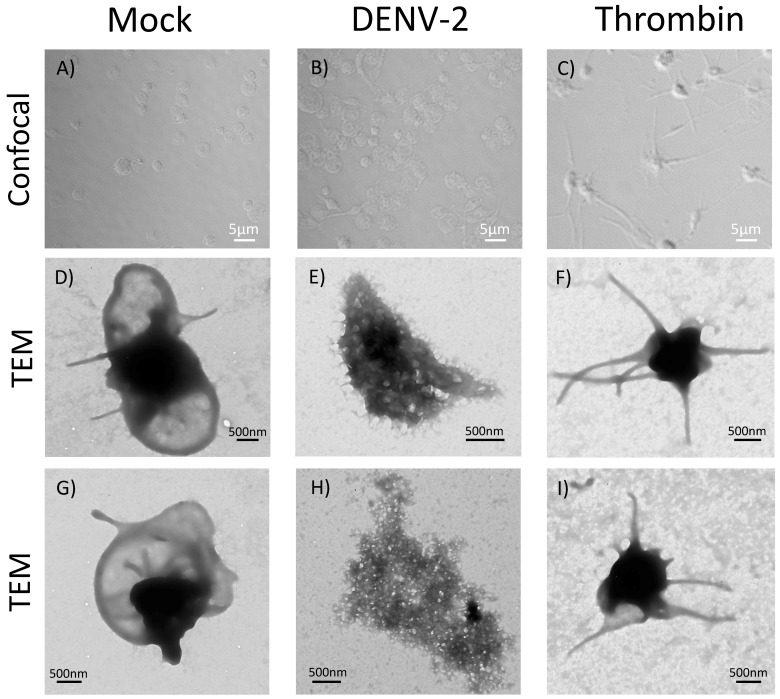
**DENV-2 induced conspicuous morphological changes in human platelets.** Platelets stimulated with DENV-2, MOI 0.2 (**B**) and MOI 0.5 (**E**,**H**) for 4 h, mock (and thrombin were incubated at 37 °C, and analyzed by TEM in triplicate. A representative image of the observed changes is presented. Conspicuous morphological changes occur in DENV-2-treated platelets (**B**,**E**,**H**), very different to those observed in thrombin positive control (**C**,**F**,**I**), meanwhile, mock control showed a normal platelet morphology (**A**,**D**,**G**). Images were captured at 50,000×. Duplicate assays were independently repeated three times.

The effect of DENV-2 stimulation on the platelet morphology was evaluated with confocal microscopy and TEM. For these assays, platelets were exposed to DENV-2 to a MOI of 0.2 for 4 h and examined by confocal microscopy; significant morphological changes were observed (Figure 6B): these included swollen and elongated cells, which were not observed in mock control (Figure 6A); platelets treated with PEG/FBS or treated with an unrelated PEG-precipitated virus. The overall analysis showed these morphological changes occurred in 31 to 35% of the platelets exposed to DENV-2 treatment at the examined MOI (0.2) and time point (4 h). In addition, notorious changes occurred in the thrombin group, although morphology clearly differed from the DENV-2-treated group. To obtain better details on the morphological changes, platelets were examined by TEM at a higher MOI (0.5) of DENV-2 at the same time point (4 h). No considerable modification, due to undesirable activation in the shape of platelets was found in the mock group (Figure 6D,G), but DENV-2 induced drastic changes (Figure 6E,H), very different to those observed in the thrombin group (Figure 6F,I). Non-stimulated platelets had a nearly discoid shape, while the thrombin control exhibited a ball-shaped platelet with a compact central electrodense zone and filopodia-like long cytoplasmic prolongations. Contrarily, platelets exposed to DENV-2 displayed multiple cytoplasmic vacuoles as well as numerous membrane blebs and microvilli (Figure 6E), meanwhile, some of these platelets displayed more profound changes (Figure 6H).

## 4. Discussion

The hallmarks of severe dengue cases are deregulated complement activation, cytokine storm, the activation and apoptosis of B and T lymphocytes, and hematologic alterations [23]. Among the fundamental features are a deep thrombocytopenia along with a vascular dysfunction. The deregulated complement system and some cytokines may contribute to endothelial damage, causing greater vascular leakage [23].

Regarding the immune response, several host cells (e.g., monocytes/macrophages, T lymphocytes and endothelial cells) participate in proinflammatory cytokine production [24]. The role of platelets in the overall immune response and vascular dysfunction has been diminished during a dengue infection. As effectors of the immune system, therefore, platelets secreted important quantities of some cytokines and are central mediators for some intercellular communication [25,26,27].

Although DENV is known to interact with platelets, it is still debatable whether the virus induces a productive infection in vitro in these cells [6,28]. However, unrefutably, platelets get infected by DENV in vivo [22,29,30]. This notorious difference is probably due to the short time (up to 6 h) that platelets can be maintained in vitro once the plasma is removed from the preparation. It is true that platelet concentrates can be conserved up to seven days; however, these showed not to be a suitable source of platelets for the kind of analysis we perform in this investigation. Clearly, in vivo analysis is both valuable and irreplaceable, but instead of allowing to assess solely the response of a single cell type, it gives information on the overall response of multiple cells participating in infection and response in a specific time point in the host. 

Another significant effect is that DENV-platelet interaction clearly causes several profound changes in cell function [16,17,18]. Two of the current findings reveal an increase in CD62P and MHC class I molecules coincide with a recent report on the proteomics of DENV-infected platelets [31]. A drop in the percentage of platelets expressing CD42b was observed for those exposed DENV in comparison with the mock group (Figure 2A). However, among those platelets treated with DENV and still positive to CD42b, the MFI revealed that the density of this molecule in the cell surface was greater than for the mock group. Thus, this could have a relevant impact at the physiological level; while some platelets may interact strongly with endothelium, others may lose this ability after being exposed to DENV. No significant differences were observed in CD42b when comparing DENV-treated (pathological) and thrombin-exposed (physiological) platelets. Thus, the pattern of CD42b found on the membrane is interesting in terms of platelet functionality. It is known that CD42b, also called GP1BA, interacts with subendothelial von Willebrand factor (vWF). Additionally, it has been linked to genetic defects leading to rare bleeding illnesses [32].

In relation to cytokines, the most significant differences between mock and DENV-treated groups were found with GM-CSF, IL-6, IL-8, IL-10, and TNF-α. It has been reported that secretion of both IL-6 and GM-CSF is significantly enhanced in patients with severe dengue. Likewise, significant systemic levels of TNF-α have been associated with severe secondary infections with dengue [33]. In supernatants, levels of three cytokines (GM-CSF, IL-6, and TNF-α) were higher than those observed with the physiological agonist thrombin. The impact of these cytokines in the overall immunopathology of dengue remains to be established in further investigations.

In regard to the expression of the CD40L protein in platelets, an almost two-fold increase was detected in the DENV-2 versus mock group at 4 h with a MOI of 0.2. The expression of the CD40L protein on the surface of platelets is known to modulate the inflammatory response of endothelial cells [34].

One of the most striking findings of the present study was in regard to the secretion of sCD40L, being much higher in platelets exposed to DENV-2 than those in the mock group. The level of sCD40L was five-fold higher at 2 h of platelet interaction with DENV-2 and two-fold higher at 4 h. Hence, platelets rapidly release sCD40L molecules upon interaction with the virus. This discovery seems particularly relevant when considering the multiple functions of sCD40L in the immune response (e.g., participation in vascular endothelium and activation of B cells). A significant role of sCD40L in both inflammation and the humoral immune response is therefore likely [5]. Giving further support to an important biological role of sCD40L in dengue is the decreased serum concentration of this molecule recently identified in patients with the virus [35].

The morphology of platelets in the DENV-2-treated group, on the other hand, was herein characterized by conspicuous changes indicative of cells in the process of dying, and observed changes seem more dramatic than in previous reports [22]. It is well documented that platelets’ apoptosis increases significantly in vivo in secondary infected patients, as well as in vitro in response to all four DENV serotypes [22]. Moreover, levels of activation (CD62P and PAC-1 binding), the presence of C3 (complement factor)/IgG on cell surface, and apoptosis (phosphatidylserine expression) correlate with drops in platelets numbers [30]. A very significant finding in both investigations is that expression of activation and apoptosis markers in platelets due to DENV exposure resulted in an increase in phagocytosis of these cells mediated by monocytic cells [22,30], which account for another key mechanism contributing to platelet destruction. 

It is important to emphasize that we used DENV MOIs trying reflected viral titers naturally attainable in an infected individual; strikingly, under these conditions, we observed significant changes in slightly more than 30% of platelets. In addition, in this experimental system, additional effectors carried in plasma (such as antibodies, complement fractions, and cytokines) were removed, allowing the sole examination of the DENV-platelet interaction. We suggest that the DENV-platelet interaction alone could account for mechanisms leading to thrombocytopenia. 

The detailed correlation of these changes with the translocation of phosphatidylserine to the platelet surface remain to be examined in future studies. However, the observed events triggered by DENV make us ponder how these may later impact platelets’ interactions with other important cellular types, including monocytes to condition phagocytosis, endothelial cells to mediate activation, and neutrophils to induce either diapedesis or production of extracellular traps. Such interactions could possibly explain some of the immunopathogenic characteristics observed with dengue infection.
